# Critical hydraulic gradient and fine particle migration of sand under upward seepage flow

**DOI:** 10.1038/s41598-022-18720-9

**Published:** 2022-08-24

**Authors:** Bo Wang, Long-wei Chen, Zhen Niu

**Affiliations:** 1 Key Laboratory of Earthquake Engineering and Engineering Vibration, Institute of Engineering Mechanics, China Earthquake Administration; Key Laboratory of Earthquake Disaster Mitigation, Ministry of Emergency Management, Harbin, 150080 Heilongjiang China; 2 State Key Laboratory for Geomechanics and Deep Underground Engineering, School of Mechanics and Civil Engineering, China University of Mining and Technology, Xuzhou, 221116 Jiangsu China

**Keywords:** Civil engineering, Sedimentology

## Abstract

In order to study the mechanism of seepage-induced geotechnical damage and characteristics of fine particle migration in sandy soil, a one-dimensional upward seepage test device was used and a series of upward seepage flow tests of sand were conducted. In these experiments, the permeability, fine particle migration and redistribution under different hydraulic gradients were investigated. The results show that local hydraulic gradient in the sand sample is larger than the critical hydraulic gradient calculated by the Terzaghi’s Equation. The seepage force will induce the fine soil particles to migrate along water flow direction and then cause the redistribution and reconsolidation of sand particles. Under the test condition, fine particles (< 0.075 mm) and fine sand particles (0.075–0.25 mm) dominate in the lost soil particles. Soil particles larger than 0.6 mm are hardly to lose.

## Introduction

Seepage-induced soil failure is involved in hydraulic and geotechnical engineering, such as earth-rock fill dam, slope, underground pipeline, foundation pit, etc. In soil mechanics, such soil failure customarily includes soil flowing and piping^1–3^. If the soil seepage failure is not effectively prevented in time, it may cause large soil deformation or even lead to failure of engineering structure. Fine soil particles may move or even become lost by seepage force, which may alter the physical and geo-mechanical characteristics of the soils (e.g., soil porosity, permeability, compressibility and strength).

It is generally believed that when hydraulic gradient exceeds the critical hydraulic gradient of soil, soil seepage deformation or failure will occur. This critical hydraulic gradient and seepage-induced soil deformation are affected by multiple factors, such as soil conditions (particle size distribution, gradation, porosity) and hydraulic conditions (seepage flow direction, hydraulic gradient)^4,5^. Kokusho et al.^6^ carried out series of constant head seepage tests on granular soils and concluded that the maximum critical hydraulic gradient for seepage failure drastically increases with increasing uniformity coefficient and becomes more than 3 times higher than the theoretical value. Rochim et al.^3^ conducted downward seepage flow test under different types of hydraulic loading. The results demonstrated that hydraulic loading history has significant effect on the critical hydraulic gradient. A long-term large hydraulic head reduces the hydraulic gradient needed for major suffusion development. Liang et al.^7^ defined a low and a high critical hydraulic gradient respectively to describe onset of the local moving and the global loss of fine particles. The test results showed that these two critical hydraulic gradients were significantly influenced by the particle size distribution and the dry density of the soil. Hunter et al.^8^ observed the movement of soil particles during seepage by camera means and found that bottom-up seepage will cause the pores of the sand sample to increase, and the fine particles will migrate upward step by step along the seepage channel, while the coarse particles as the skeleton will conduct an internal rearrangement. Ueng et al.^9^ analyzed the change of soil permeability characteristics in the seepage process through a one-dimensional upward seepage test of constant hydraulic head of sand sample and found that the permeability coefficient of the sample will increase by 4–5 times under the action of upward seepage force inside the sample, and it will recover after the seepage stops. Koltuk et al.^10^ carried out a series of two-dimensional steady-state numerical and experimental water flow tests to investigate the quicksand condition. The results showed that the ratios of theoretical and experimental value of quicksand condition varied between 0.8 and 1.1 for loose and medium-dense sands.

For the phenomenon of the migration or loss of fine particles inside the soil under the action of seepage flow. In the literature, it can be found that when the seepage effect is small, the soil particles will not migrate and the permeability coefficient remains unchanged. With an increase of the seepage effect, the soil will produce seepage deformation, and the permeability coefficient will gradually increase. Thus, it is considered that fine particle migration is one of the necessary conditions for the change of the permeability coefficient of cohesionless soil. For soil with a specific structure, only fine particles within a range of certain size could be eroded and lead to changes in porosity. Some of the migrated particles will be lost and the others with larger size will settle down during the seepage process. Wang et al.^11^ analyzed the migration of soil particles under seepage based on the theory of the sphere hypothesis. Starting mode of soil particle migration was divided into rolling and sliding types and the corresponding critical hydraulic gradient formula were given. Wu et al.^12^ proposed that upward seepage-induced fine particle migration includes two starting mode, one is the mode of single fine particle and another one is the mode of fine particle group. The starting mode of fine particle group was closer to the experimental result than that of the single fine particle. Based on the literature, it can be seen that the soil seepage failure mode and particle transport characteristics are complex and affected by many factors. This paper uses a self-made one-dimensional upward seepage test device to carry out sand seepage tests under different hydraulic gradient conditions, and focus on variation of soil permeability and laws of fine particle migration caused by seepage flow. These results have a reference value for the analysis of engineering soil seepage stability problems.

## Test device and method

### Test device

**Figure 1:**
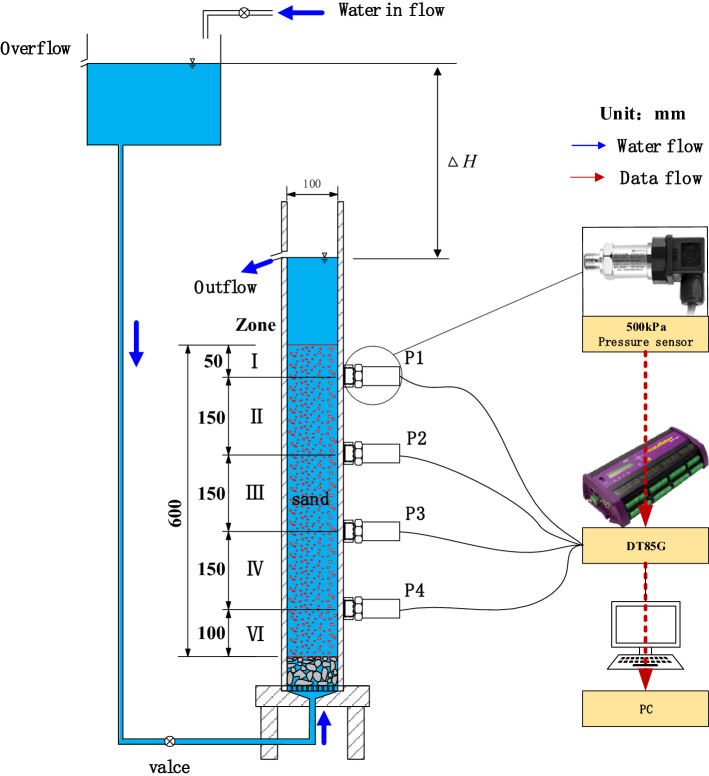
Sketch of test device.

In order to observe the behavior of sand particles, the seepage test device is made of transparent acrylic material. As shown in Fig. 1, the seepage test device mainly includes four components, namely, a hollow cylinder with an inner diameter of 100 mm, a water channel, support and sensors. Four pore water pressure sensors are installed on the hollow cylinder with equal spacing. Data of these sensors are recorded in real time by DT85G acquisition instrument. Position of the overflow outlet of the water channel is higher than the hollow cylinder. Thus, a whole constant hydraulic head will be applied in the hollow cylinder to initiate the upward seepage flow. The upward seepage flow will reduce the effective stress between soil particles. In this paper, this condition is referred as low effective stress state. As the upward seepage force continues to increase and exceeds the effective weight of soil, the soil particles will be in a state of weightlessness or suspension. In this condition, soil particles no longer contact each other and can be considered in zero effective stress state. Therefore, different upward seepage force corresponds to different effective stress state of soil and this low effective stress state in specimen can be judged by the measured pore water pressure during test.

According to the arrangement of the pore water pressure sensors, the sand specimen can be divided into five zones, from zone I to zone V. Data of pore water pressure sensors P1–P4 are recorded as *u*_1_–*u*_4_. The hydraulic gradient of zone II to zone IV in the sand specimen can be calculated as follows:

1 $$i_{i,j} = \frac{{\Delta h_{i,j} }}{{L_{i,j} }} = \frac{{u_{j} - u_{i} }}{{\gamma_{w} L_{i,j} }} - 1$$

where ∆*h*_*i,j*_ is the hydraulic head difference acting on the zone (cm), $L_{i,j}$ is the corresponding height of the sand (cm) and $\gamma_{w}$ is the gravity density of water.

Further, the average permeability coefficient of sand in zones II to IV can be estimated as follows:

2 $$k_{i,j} = \frac{Q}{{A \cdot t \cdot i_{i,j} }}$$

where *Q* is the amount of water flow out of sand sample (ml), *A* is the cross-sectional area of sand sample (cm^2^), and *t* is the time (s).

### Test method

**Figure 2:**
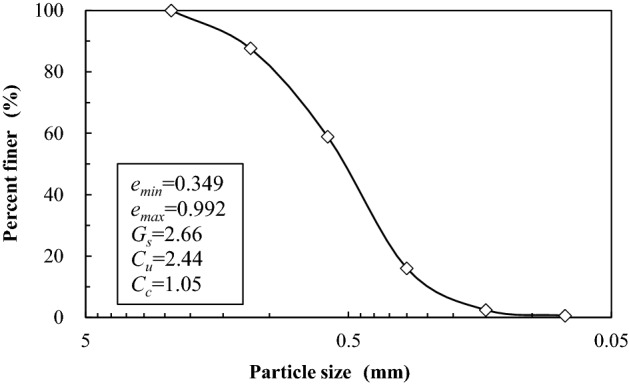
Grain size accumulation curve of the test sand.

The sand used in seepage test is coarse sand. Particle grading and basic parameters of the coarse sand are shown in Fig. 2. The fine particle content (particle size less than 0.075 mm) in the coarse sand is about 0.6%.

The sand specimen is filled underwater layer by layer. It is about 18 layers in total and the sand weight for each layer is about 0.42 kg. In order to ensure the accuracy of sand filling, a measuring tape is pasted on the outer wall of the container and a tamping rod is used to compact the filled sand to reach the predetermined height. After the last filling, the surface of the sand sample is gently smoothed and water is slowly purged into the cylinder. This process takes about 4 h to ensure the quality of sampling. The height of the filled sand sample is 600 mm, the specific gravity of the filled sand is 44.7%, and the corresponding void ratio is 0.704.

The procedure of constant hydraulic head seepage test in this paper is as follows:

1. Before the test, slowly add water from the upper part of the sample container to the overflow hole, and continuously read the pore pressure sensor data. By comparing with the hydrostatic pressure value to verify that whether the pore pressure sensor and acquisition system work normally and reliably.
2. Open the water inlet valve of the water channel to keep the water surface at the designed height (excess water flows out of the water channel overflow). Then, open the water stop valve between the water channel and the cylindrical sand sample. Under the action of the hydraulic head drop, the water will flow through the sand sample from bottom to top and then flow out from the overflow at the top of the cylinder. At this time, upward one-dimensional seepage flow is formed inside the sand sample, and the water flowing out of the cylinder (used to calculate the average seepage process sample permeability coefficient) is collected at a certain time interval. Overflow water by standing. The overflow water is placed, water and soil are separated, and the soil sample is dried and weighed.
3. Close the water stop valve between the water channel and the cylindrical sand sample. The soils begin the process of reconsolidation. The data of the pore pressure sensor continue to be collected. Carefully observe the changes of the sand sample and recorded them with a camera.
4. After variation of the pore water pressure is stable, the constant hydraulic head test is carried out on the reconsolidated sand sample. The water flowing out of the bottom of the sand sample at a fixed time interval is recorded (used to calculate the permeability coefficient of the reconsolidated sand sample).
5. After the end of the constant hydraulic head test, the water in the cylinder was drained and sampled according to the sections as shown in Fig. 1. After drying, the grading of sand samples in zones I–V after seepage test was obtained by sieve analysis method.
6. Adjust the height of the water channel and the cylindrical overflow port of the sand sample (change the whole hydraulic head difference), refill the specimen, and repeat steps 1–5 to complete the next set of tests.

A total of 5 groups of tests were conducted in this paper, and the specific arrangement is shown in Table 1.

**Table 1:** Test arrangement.

Index	∆*H*/cm	Average *i*
T1	154	2.567
T2	139	2.317
T3	124	2.067
T4	109	1.817
T5	95	1.583

## Results and analysis

### Critical hydraulic gradient of the sand

**Figure 3:**
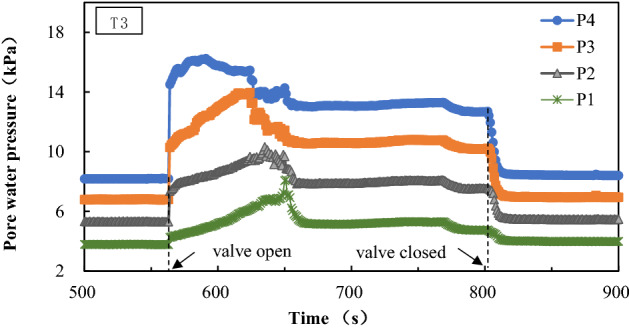
Variation curve of pore water pressure in T3.

According to the experimental phenomenon and analysis of the collected pore water pressure data (as shown in Fig. 3), the seepage in the test process can be divided into three stages:

1. Seepage steep increase stage: After the water stop valve is opened, the hydraulic head difference acts on the sand sample, and the pore water pressure increases rapidly on the basis of hydrostatic pressure. Through the test device, it can be observed that there are some seepage flow channels in the upper part of the sand samples, accompanied by the upward migration of fine particles.
2. Stable seepage stage: After the pore water pressure reaches the peak value, there will be a relatively rapid decline and oscillating rise, finally entering a relatively stable state. That is, the sand sample enters a low effective stress state under the action of stable seepage. At this stage, it can be observed that the middle and upper sand samples boil, and the soil particles will continue to overflow with the seepage process.
3. Reconsolidation stage: After closing the water stop valve, the pore water pressure in the sand sample will recover to the hydrostatic pressure level quickly, suspended soil particles will gradually settle, and the soil sample structure will be reconstructed. This is the reconsolidation stage of sand sample. After reconsolidation, a fine-grained layer is deposited at the top of the sand sample and the soil particles are clearly distributed from fine to coarse from top to bottom.

Based on soil mechanics, we can use the classic Terzaghi formula to evaluate the critical hydraulic gradient of soil.

Tazaghi’s equation

3 $$i_{cr} = \frac{{\gamma_{s} - \gamma_{w} }}{{\gamma_{w} }}\left( {1 - n} \right) = \frac{{G_{s} - 1}}{1 + e}$$

**Figure 4:**
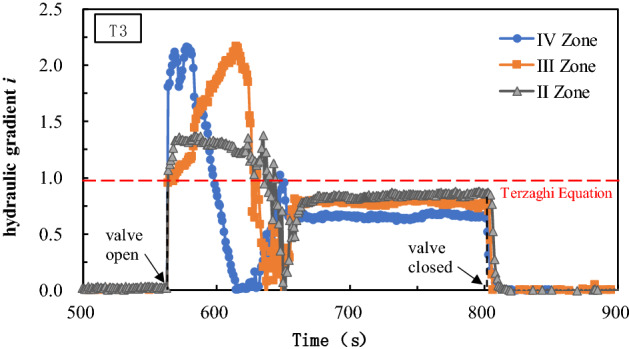
Variation curve of the hydraulic gradient in T3.

According to Tazaghi`s equation, the critical hydraulic gradient of the sand used in this paper is 0.97. By substituting pore water pressure data into Eq. (1), hydraulic gradient values acting on the sand samples in zones II to IV during the test can be calculated, as shown in Fig. 4. It can be seen that the hydraulic gradient in the sand samples from zone II to IV will generate a peak value before reaching a stable seepage state, which corresponds to the critical hydraulic gradient that triggers seepage failure. The measured critical hydraulic gradient is about 1.5–2.0 times of the value calculated by the Terzaghi`s equation. There are some differences between the theoretical calculation and the experimental results. This may be related to some factors, such as the accuracy of the basic parameters of the sand sample and the friction of the side wall of the test vessel. After the peak value, the hydraulic gradient goes through the trough and oscillates and then enters the relatively stable seepage stage. The hydraulic gradient in the stable stage is close to the calculation result of the Terzaghi’s equation.

**Figure 5:**
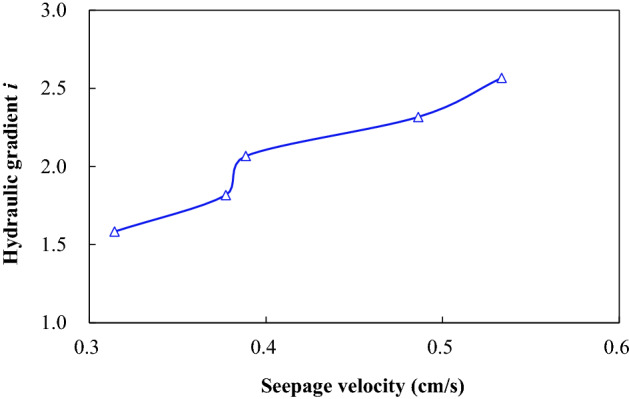
Variation curve of the hydraulic gradient and seepage velocity.

During the period starting from when the water stop valve is opened to the sand sample reaching a stable seepage, the hydraulic gradient has gone through a process of increasing—decreasing—increasing again, which indicates that a large seepage force is needed to destroy the soil particle structure before seepage failure occurs. From the local seepage to the channel interconnection, the seepage failure of soil particle structure is manifested by a rapid decrease of pore water pressure. However, due to the different thicknesses of the overlying soil layer and the initial soil structural influence, the pore water pressure peaks and decreases at different locations are not the same. The test results show that the critical hydraulic gradient peak of the deep soil is greater than that of the shallow one, and the fluctuation range of the hydraulic gradient is larger than that of the shallow part. The overall performance is that as the thickness of the overlying soil layer increases, it is more difficult to trigger soil seepage failure. From the test, we can conclude that the soil seepage failure occurs gradually from shallow to deep. Figure 5 shows the variations of seepage flow velocity and hydraulic gradient in test T1–T5. Under test conditions, the seepage flow velocity increases with increasing hydraulic gradient. It needs to be pointed out that the flow velocity and the hydraulic gradient in Fig. 5 are average values.

### Migration characteristics of fine particles

**Figure 6:**
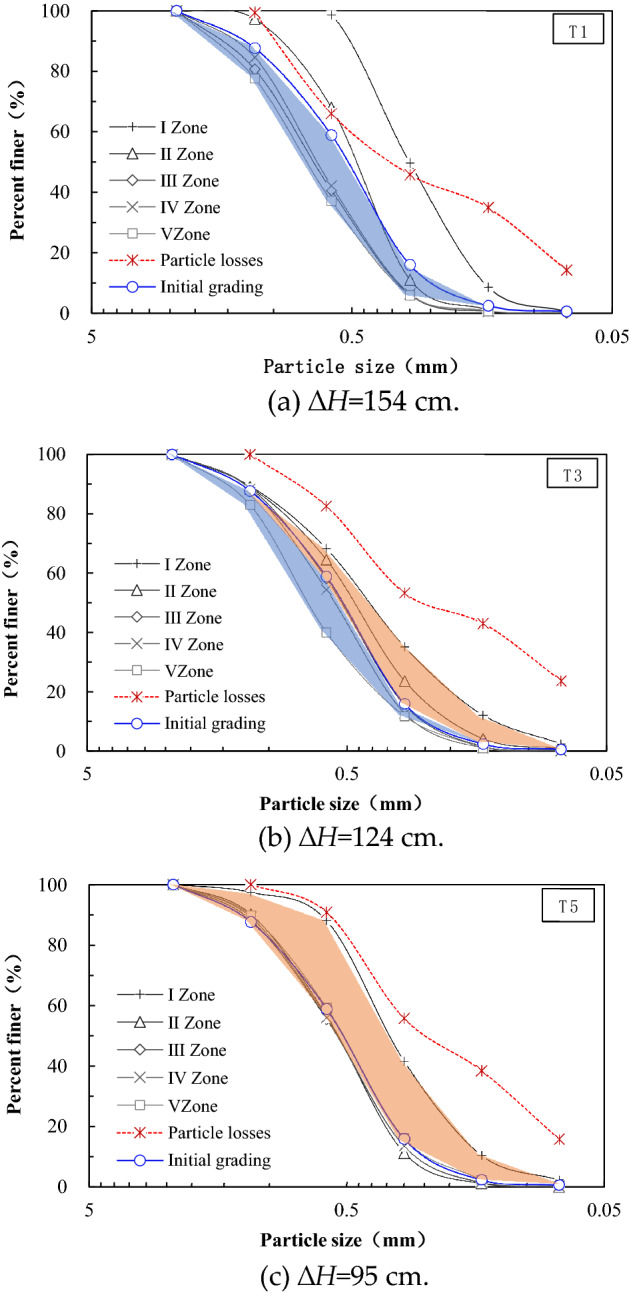
Grading curve of sand samples in each sand zone.

An obvious fine particles migration phenomenon was observed in the test. As shown in Fig. 6, fine particle sand is easily washed away and lost by water flow under the action of upward seepage, and with the increase of hydraulic head drop, the fine particle loss caused by seepage becomes more serious.

To be specific, the grading of sand samples in zones I–II after the test move significantly to the right compared with the initial curve. That is, the soil particle size became smaller. The gradation curves of sand samples in zones III–V move the left compared with the initial curve. But the three curves are overlapped with the decrease of the hydraulic head drop. Under the action of upward seepage force, the shallow soil particles will firstly out of contact and the seepage path becomes wider. The finer and lighter particles will move upward to the surface along the seepage path. Some fine soil particles in the local area of the surface layer will follow the water and loss. At the same time, coarser and heavier particles tend to sink, and finally the entire sand sample exhibits a fine-to-coarse particle size distribution from top to bottom.

Wang et al.^13^ simulated the migration process of fine particles under the action of seepage through PFC model. If the sand sample is divided into five layers from top to bottom, it is found that the number of fine particles in the bottom fourth and fifth layer will continue to decrease and supplement the middle second and third layer, and eventually the number of fine particles in the layer change became smaller, while the inflow of fine particles in the first layer was greater than the loss of the layer, leading to a gradual increase in the number of fine particles in this layer. The above research results are basically consistent with the observed phenomena in this paper. Fine particles (< 0.075 mm) and fine sand particles (0.075–0.25 mm) dominate in the lost soil particles, and there are few sand particles with a particle size greater than 0.6 mm. Under the seepage condition in this paper, soil particles smaller than 0.15 mm are easy to lose, and soil particles larger than 0.6 mm are hardly to lose.

**Figure 7:**
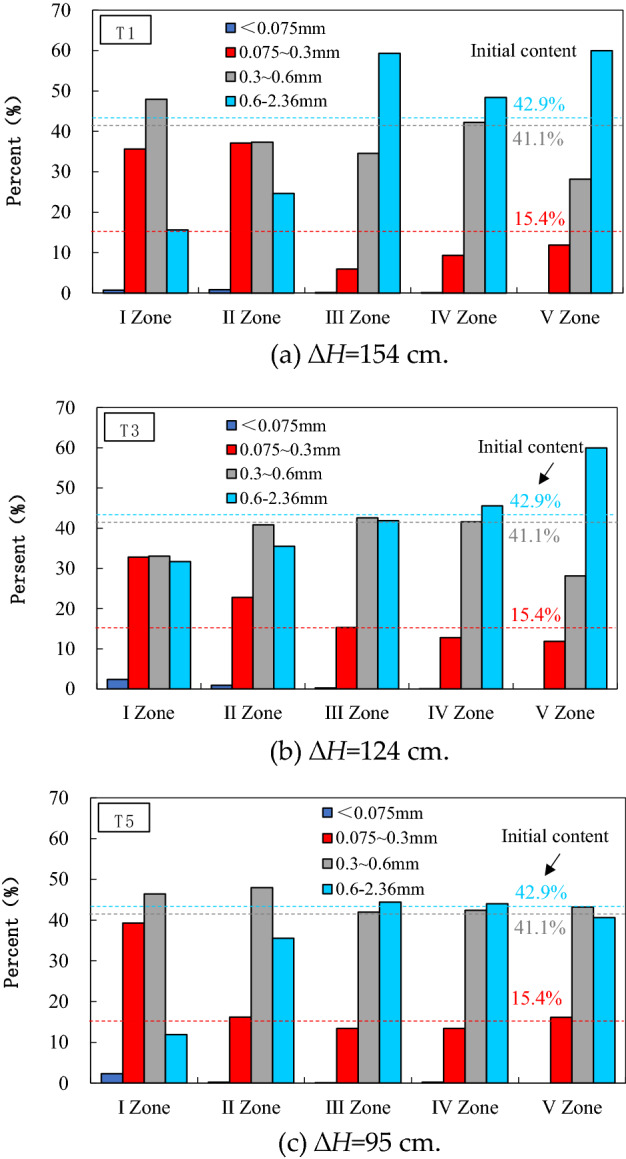
Variation of soil particles with hydraulic head in different sand zones.

As shown in Fig. 7, the range of particle content increased in zones I–II is 0.075–0.3 mm, and the range of particle content increased in zone III–V is 0.6–2.36 mm. There is little change in soil weight content of 0.3–0.6 mm in sand samples from zone I–V. In other words, considering the soil layers from top to bottom, the particle size range of the increased content gradually increased, which means that fine particles migrate upward, while coarser particles deposit downward. The seepage effect will cause the redistribution of soil particles and the reconstruction of the particle structure inside the sand sample.

After the test, height of the sand sample increases about 10–25 mm compared with the initial height. Considering that part of the fine particles will be deposited on the top of the sand sample, these deposited fine particles will increase the volume of the sand sample to a certain extent. On the other hand, the lost fine particles will cause the volume of the sand sample to decrease. In these tests, the weight of the lost soil particles is about 1.8–3.8% of the total. The fine particles that originally filled in the pores of the coarse particles migrate and deposit on the top to increase the volume of the sand sample, while the loss of fine particles does not cause a comparative volume reduction of the larger coarse particle framework. Therefore, the overall volume of the sand sample is increased slightly after seepage.

## Conclusions

A one-dimensional upward seepage test device was used and a series of seepage tests of sand were carried out; the main conclusions are as follows:

1. Seepage failure initiation: Under upward seepage flow, the soil seepage failure occurs gradually from shallow to deep. As there are dense arrangement of sand particles and fewer seepage paths, seepage flow needs a certain amount of strength and time to break through the connection force of the sand particles. In this case, the peak of seepage force may occur and then the sand enters a stable seepage state.
2. Critical hydraulic gradient: Test results show that the measured local hydraulic gradient in the sand sample is about 1.5–2.0 times of the critical hydraulic gradient calculated by the Terzaghi’s Equation.
3. Fine particle migration: Under the action of upward seepage force, lighter fine particles will migrate upward, and heavier coarse particles will deposit downward. Phenomenon of particle redistribution and structure reconstruction will occur.

## Data Availability

The datasets used and/or analysed during the current study available from the corresponding author on reasonable request.
